# Thyrotropin reference interval in older adults

**DOI:** 10.1530/EC-24-0435

**Published:** 2025-01-02

**Authors:** Xiaodan Zhai, Yongze Li, Xiaochun Teng, Weiping Teng, Xiaoguang Shi, Zhongyan Shan

**Affiliations:** ^1^ Department of Endocrinology and Metabolism, Institute of Endocrinology, National Health Commission Key Laboratory of Diagnosis and Treatment of Thyroid Diseases, The First Hospital of China Medical University, Shenyang, Liaoning, P. R. China; ^2^ Department of Endocrinology, Shengjing Hospital of China Medical University, Shenyang, Liaoning, P. R. China

**Keywords:** thyrotropin, reference interval, older adults, hypothyroidism

## Abstract

It is estimated that by the year 2050, 16% of the world’s population will be 65 years old and above. As the global aging population continues to grow, there is an increasing focus on thyroid disorders among older individuals. Thyrotropin is widely used in diagnosing subclinical thyroid diseases due to its high sensitivity as an indicator of changes in thyroid function. However, thyrotropin levels change with age, and different reference intervals have been proposed in various studies. The variation in thyrotropin ranges among older adults is probably caused by the heterogeneity of the studied population. This review aims to provide an overview of the existing literature on thyrotropin reference intervals in older adults and their distinction as adaptive or pathologic. Recent research indicates that older individuals may have slightly elevated levels of thyrotropin and higher upper limits of reference intervals. Therefore, a higher thyrotropin threshold for diagnosing and treating subclinical hypothyroidism in the elderly seems reasonable.

## Introduction

As life expectancy increases, the proportion of older adults continues to rise globally. Thyroid disease is a common condition among older people, with subclinical hypothyroidism (SHypo) being the most common form of the disease (1). SHypo is characterized by elevated serum thyrotropin (TSH) levels and normal free thyroxine (FT4) levels. Meanwhile, subclinical hyperthyroidism (SHyper) is characterized by decreased serum TSH levels and normal FT4 levels. The pituitary gland is highly sensitive to changes in thyroid function, which can result in a six-fold increase in TSH levels when daily thyroxine (T4) production decreases by 30% (2). Therefore, despite not being perfect, the serum concentration of TSH remains the most sensitive indicator of changes in thyroid function. However, serum TSH levels tend to increase with age, particularly in women (3, 4). Despite the higher positivity rate of autoimmune thyroid antibodies in older adults, the TSH levels remain higher than those in young adults when individuals with positive antibodies are excluded (5).

Aging primarily impacts the upper limit of the TSH reference interval (RI). After the age of 40, the upper limit of the serum TSH RI increases by 0.3 mIU/L for every 10-year increase in age (6). However, the concentration of FT4 remains unchanged during healthy aging (7). Basic studies have demonstrated that the thyroid gland takes up less iodine, and the thyroid weight, colloid component and follicular volume decrease in the older population. As a result, older adults produce fewer hormones in the thyroid gland. Due to the slow degradation rates, serum FT4 levels remain constant (8). Serum TSH levels increase in older adults, presumably due to a reduction in TSH bioactivity or a decreased responsiveness of the thyroid to TSH (9). The increase in serum TSH levels in older people without a reduction in circulating FT4 may indicate a phenomenon of physiological adaptation. This brief review aims to first summarize the existing literature on TSH intervals in older adults and their interpretation as adaptive or pathologic.

## Methods

We conducted a narrative review of the literature and searched PubMed using keywords such as thyroid function, thyrotropin, TSH, reference range, RI, older, aging and elderly. We reviewed relevant articles published in English. The definition of older adults lacks a clear age cutoff, and the criteria used in the articles we reviewed were inconsistent.

## Influencing factors and measurement of thyrotropin

### Genetics

An individual’s serum TSH level is largely determined by genetics (10, 11). In healthy individuals, the individual TSH reference ranges were narrower, approximately half of those defined based on group values (12). A genetic study of an Ashkenazi Jewish population reported that higher TSH concentrations were associated with the presence of two single nucleotide polymorphisms (SNPs) in the promoter/enhancer region of the TSH receptor gene. The frequency of the two SNPs was also higher in centenarians and associated with exceptional longevity (13). It may provide a basis for the increase in TSH levels with aging.

### Thyroid autoimmunity

Despite the higher positivity rate of autoimmune thyroid antibodies in older adults, TSH levels remain higher than those in young adults when individuals with positive antibodies are excluded (5). It is believed that thyroid autoimmunity alone may not be the cause of elevated TSH levels in older individuals. On the other hand, lymphocytic infiltration of thyroid tissue was found in approximately half of older individuals (14). However, the positive rate of circulating thyroid autoantibodies does not reach 50% in older adults (5, 6). The discrepancy suggests that some individuals with lymphocytic thyroiditis may not have detectable circulating autoantibodies. Since thyroid autoimmunity is the main cause of thyroid dysfunction, it is crucial to carefully exclude patients with thyroid autoimmunity when selecting a reference population expected to have truly normal thyroid function. Therefore, researchers recommend using thyroid ultrasound when selecting a reference population to exclude subjects with diffuse thyroid diseases (15).

### Iodine nutritional status

The iodine nutritional status of the populations studied may result in different thyroid function patterns. A moderate to severe iodine deficiency can cause thyroid dysfunction due to insufficient substrate for thyroid hormone production. Conversely, high iodine intake can lead to increased levels of TSH, even in children who are less likely to have thyroid autoimmunity compared to adults (16). With the increase in urinary iodine concentration (UIC), serum TSH levels tended to increase (17, 18). Participants older than 70 years had the highest UICs (median: 442.5 μg/L) in a Korean population (17). In populations with mild iodine deficiency, the serum level of TSH was negatively associated with age, whereas an ongoing decrease in TSH was observed following iodine supplementation (19). Therefore, it is proposed that both the present and historical iodine status of a population should be taken into account to accurately establish the RIs of TSH (20).

### Glycosylation and immunoassay interference

Pituitary TSH is heterogeneous in terms of glycosylation levels (21), resulting in differences in antibody binding and biological activity (22). However, changes in glycosylation with age have not been studied. On the other hand, immunoassay platforms are currently the method for the measurement of thyroid function tests. Interference from macro-TSH, biotin, thyroid hormone autoantibodies and heterophilic antibodies is common (23). Consequently, it is important to establish RIs for each TSH assay using varied antibodies (24).

### Circadian rhythm and sleep

TSH is secreted from the pituitary gland in pulses, causing fluctuations in serum concentration. These pulses occur more frequently at night, with TSH levels being 50% higher during the nighttime compared to the daytime (25). Sleep withdrawal, which is more common in older people, augmented the nightly TSH secretion (25). It has also been reported that there is a 1- to 1.5-h shift in the circadian rhythm of TSH secretion in older subjects, resulting in an earlier peak (26, 27). Therefore, TSH levels are influenced by the time of blood collection and the sleep status of the subjects. It is recommended that an initially elevated serum TSH be investigated with a repeat measurement, preferably after a 2- to 3-month interval (28).

### Non-thyroidal illness, medication and obesity

In individuals with severe illness, TSH levels can be lower than normal due to the adaptation of the hypothalamic–pituitary–thyroid axis to the illness or medications such as glucocorticoids or dopamine. During recovery from illness, TSH levels may increase transiently above the normal range (29). Obesity and overfeeding are often accompanied by increased TSH as a result of oversecretion of leptin (30).

### Thyrotropin RIs in older adults

According to Guideline 22 of the National Academy of Clinical Biochemistry (NACB), a reference population was selected after excluding individuals with positive thyroid antibodies, a personal or family history of thyroid dysfunction, thyroid goiter or nodules that were distinguishable or palpable and the use of thyroid medications (24). The RI for TSH is calculated as the range between the 2.5th and 97.5th percentiles of serum TSH levels from a large group of selected individuals (24). The TSH RIs in older reference populations in various studies are summarized in Table 1. In some clinical studies with large samples, the results were based on disease-free populations who did not report any thyroid diseases, goiter or taking thyroid medications, without being strictly selected according to the NACB criteria.

**Table 1:** Summary of the thyrotropin RIs in older reference population.

No.	Region	Author, year	Data source	Iodine status	Manufacturer	Subjects	Gender (men: women)	Main finding(s)
1	Germany	Völzke (2005) (15)	Epidemiologic survey	Previously ID, currently IS	Byk Sangtec	1,488; 190 older adults (60–79 years)	1:0.8	TSH levels decrease with age and are significantly lower than those established in areas with IS Presented as median (interquartile range) 60–69: 0.66 (0.49) in males, 0.60 (0.75) in females 70–79: 0.69 (0.50) in males, 0.65 (0.62) in females Total: 0.25–2.12
2	Australia	O’Leary (2006) (31)	Epidemiologic survey	IS	DPC	2,026; 468 older adults (≥60 years)	1:1	60–69: 0.47–4.10 in males, 0.48–6.25 in females ≥70: 0.51–5.33 in males Total: 1.25 (0.4–4.0)
3	China	Guan (2008) (18)	Epidemiologic survey	Different iodine statuses	-	2,237; 100 older adults (≥60 years)	1:2.5	≥60: 1.40 (0.34–4.74) Total: 1.44 (0.31–4.78)
4	Japan	Takeda (2009) (32)	Outpatient	-	Roche	857; 140 older adults (≥ 60 years)	1:0.9	TSH was positively correlated with age 60–69: 2.01 (0.57–4.75) ≥70: 2.17 (0.75–5.37) Total: 1.65 (0.51–4.57)
5	Japan	Yoshihara (2011) (33)	Outpatient	MTA	Roche	1,388; 121 older adults (60–80 years)	1:3.3	The upper limit of the normal range of serum TSH increased with age Presented as (−2SD, mean, +2SD) 60–69: (0.60, 2.10, 4.85) in all, (0.22, 2.68, 5.14) in males, (0.71, 1.93, 5.25) in females ≥70: (0.63, 1.96, 6.15) in all, (0.52, 1.48, 4.23) in males, (0.83, 2.39, 6.88) in females Total: (0.44, 1.48, 4.93)
6	China	Li (2011) (34)	Epidemiologic survey	IS	DPC	2,488; 349 older adults (≥60 years)	1:1.6	The TSH levels of women were higher than those of men in the same age group 60–69: 1.35 (0.42–7.57) in males, 1.53 (0.48–5.34) in females 70–79: 1.22 (0.17–4.53) in males, 1.43 (0.63–5.56) in females 80–85: 1.30 (0.47–2.51) in males, 1.52 (0.54–4.31) in females Total: 1.45 (0.46–5.46) in males, 1.68 (0.50–5.52) in females
7	India	Marwaha (2013) (35)	Epidemiologic survey	IS	Roche	1,916; 437 older adults (≥61 years)	1:1.1	Presented as median TSH 61–70: 2.00 in males, 2.12 in females ≥71: 1.90 in males, 1.96 in females Total: 1.60 in males, 1.74 in females
8	Iran	Amouzegar (2013) (36)	Epidemiologic survey	IS	Roche	2,199; 381 older adults (≥60 years)	1:1.3	≥60: 1.34 (0.26–4.95) Total: 1.46 (0.32–5.06)
9	Thailand	Sriphrapradang (2014) (37)	Epidemiologic survey	IS	Roche	1,947; 903 older adults (≥60 years)	1:0.8	The upper normal limit of TSH tended to increase with age 60–69: 1.64 (0.43–5.2) 70–79: 1.55 (0.31–5.18) ≥80: 1.72 (0.22–6.53) Total: 0.34–5.11
10	Korea	Kim (2015) (38)	Health checkups	IE	DiaSorin S.p.A	5,778; 1117 older adults (60–79 years)	1:0.5	60–69: 2.42 (0.67–8.69), 2.35 (0.66–7.30) in males, 2.70 (0.72–9.99) in females 70–79: 2.37 (0.57–9.70), 2.26 (0.53–9.64) in males, 3.0 (1.0–8.98) in females Total: 2.38 (0.72–7.79)
11	China	Cai (2016) (39)	Epidemiologic survey	Previously ID, currently MTA	Siemens	717; 185 older adults (61–85 years)	1:1.2	61–70: 1.49 (0.39–5.68) ≥71: 1.65 (0.52–5.20) Total: 1.54 (0.43–5.51)
12	Korea	Kwon (2017) (40)	Epidemiologic survey	IE	Roche	Older adults (≥60 years)	1:0.9	The TSH levels of women were higher than those of men in the same age group 60–69: 2.20 (0.57–6.90), 2.11 (0.55–6.55) in males, 2.27 (0.57–7.17) in females ≥70: 2.28 (0.42–6.58), 2.02 (0.28–6.14) in males, 2.53 (0.42–6.82) in females Total: 2.23 (0.62–6.84)
13	China	Zhai (2018) (5)	Epidemiologic survey	IS or MTA	Roche	8,041; 747 older adults (65–92 years)	1:1.4	≥65: 2.58 (0.75–8.86) Young: 2.38 (0.76–6.57)
14	China	Zhao (2020) (41)	Epidemiologic survey	IS or MTA	Roche	58,684; 9297 older adults (65–92 years)	1:1.1	Increased age was associated with an increased TSH 97.5th centile 60–69: 2.31 (0.64–8.19) ≥70: 2.45 (0.6–10.50) Total: 2.28 (0.74–7.04)
15	China	Ni (2022) (42)	Epidemiologic survey	MTA	-	1,074 older adults (≥65 years)	-	The median level and upper limit of TSH increased with age 65–70: 2.06 (0.65–5.51) 71–80: 2.27 (0.85–5.89) ≥81: 2.66 (0.78–6.70)
16	China	Lu (2023) (43)	Health checkups	IS	Abbott	1,109; 261 older adults (50–70 years)	1:0.7	50–70: 1.90 (0.64–5.48) in males; 2.49 (0.54–5.17) in females Total: 0.70–4.93

TSH, thyrotropin; UIC, urinary iodine concentration; ID, iodine deficient (UIC < 100 μg/L); IS, iodine sufficient (UIC 100–199 μg/L); MTA, more than adequate (UIC 200–299 μg/L); IE, iodine excess (UIC ≥ 300 μg/L); RI, reference interval.

The RIs for TSH are presented as median (2.5th–97.5th percentile) (mIU/L), and some of the results are not shown if absent.

Several changes in thyroid function occur with advancing age. In the past, most studies demonstrated an age-dependent decline in serum TSH levels (44). TSH was inversely associated with age (45, 46), a trend that has also been reported in populations with previous iodine deficiency (15, 19). Another potential reason could be an age-related decrease in pituitary TSH secretion (47). Several studies have reported a similarly low TSH response to low levels of thyroid hormone in older patients with overt hypothyroidism (48, 49). Other mechanisms, such as reduced secretion of hypothalamic thyrotropin-releasing hormone, cannot be excluded. However, the Whickham study conducted in the UK in 1977 found that there was no significant relationship between TSH levels and age. This could partly be attributed to the use of less accurate TSH assays (50). Three longitudinal studies did not confirm age-related changes in TSH levels, which may be attributed to the shorter follow-up periods in these studies (4, 51, 52).

More recently, most studies have demonstrated an increase in the upper limit of serum TSH RIs (3, 6, 53, 54). Longitudinal studies have also shown that aging is associated with an increase in TSH concentrations (9, 55). There was a 13% increase in TSH (55) or a mean TSH change of 0.32 mIU/L (9) over the 13-year period. Although the exact mechanism is unclear, the increase in TSH levels in older individuals may be a normal phenomenon that is associated with the aging process. Between 1981 and 1994, researchers in Australia conducted a study and discovered that participants who had the lowest TSH levels at baseline showed the most significant increase in TSH over time. This indicates that the rise in TSH levels may be due to age-related changes in the TSH set point or decreased TSH bioactivity, rather than occult thyroid disease (9). It has also been postulated that the thyroid may be less sensitive to TSH in older adults (56). There are insufficient *in vivo* human data to support either hypothesis.

Studies have shown that the TSH RI widens as age increases, with lower 2.5th percentiles observed in older age groups (6, 54). Data from the UK have indicated that the 2.5th percentile of TSH levels decreased with age, ranging from 0.51 to 0.31 mIU/L (54). The occurrence of mild hyperthyroidism also increases in older adults, especially in populations with a history of or current iodine deficiency. In other studies, it has been observed that the normal distribution curve of TSH shifts to the right in older individuals (3). Failure to use robust criteria to exclude patients with thyroid nodules and positive thyroid antibodies may result in inconsistency (3).

Even among elderly individuals, TSH RIs can vary between different age groups. Most studies have demonstrated a higher upper limit of serum TSH RIs in the age group of 70 years and above compared to the age group of 60–69 years (31, 32, 33, 37, 38, 41), especially in females (33). The RIs for serum TSH also vary among older individuals of different genders and races (53, 57). The Whickham survey revealed a significant increase in TSH levels in women aged >45 years, while no such trend was observed in men (50). Women often had higher TSH levels compared to men in the same age group (34, 58). Table 1 summarizes the results from various regions, also indicating the discrepancy in TSH RIs among different ethnicities.

There are some results from disease-free populations that are not summarized in Table 1 but reached similar conclusions. In the NHANES survey, the upper limits of TSH progressively increased with age (3). The 97.5th percentile of TSH also significantly increased with age in Australia (59) and the United Kingdom (54). However, some studies did not demonstrate the increasing trend (60, 61, 62). The inclusion of individuals with antibody positivity and variations in iodine status could potentially affect the results and TSH ranges in these studies. However, it was reported that serum TSH levels were higher in white non-Hispanics (whites) than in black non-Hispanics (blacks) or Mexican Americans, and elevated TSH levels (TSH >4.5 mIU/L) were more common among whites (6). Analyzing results from disease-free populations can provide an insight into prevalence and racial differences.

It has been suggested that the current upper limit of the TSH RI is often too low for older adults. The 2012 American Thyroid Association/American Association of Clinical Endocrinology guidelines for hypothyroidism in adults recommended that the normal TSH reference range may widen with increasing age (63). The 2013 ETA guidelines for SHypo recommended that age-specific reference ranges for TSH should be considered to establish a diagnosis of SHypo in older people (28). Until June 2020, the French Society of Endocrinology (SFE) released the first special consensus on thyroid dysfunction for the elderly population worldwide (64). It is recommended that the lower limit of the TSH range is usually 0.4 mIU/L and is relatively unaffected by age. In subjects over 60 years old, the patient’s age (decade) divided by 10 should be used to determine the upper limit of the TSH range. For example, 7 mIU/L is used for those aged 70–79 years and 8 mIU/L for those over 80 years (64). Many older patients with elevated TSH levels may experience stable conditions or even recovery of their condition without any intervention (65). Therefore, it is recommended that diagnosis and treatment be based on at least two tests (28, 64).

Due to a lack of large-scale multicenter prospective studies, there is uncertainty regarding the clinical benefits of treating elderly patients with mild SHypo (66). Several cross-sectional studies and meta-analyses failed to find an increased risk of metabolic diseases, cardiovascular events or mortality in older adults with slightly elevated TSH levels (67, 68, 69). Therefore, the 2017 Chinese guidelines recommended treating elderly patients with severe SHypo (TSH ≥10 mIU/L). The 2020 SFE guidelines recommended levothyroxine replacement therapy when TSH levels are greater than 20 mIU/L in at least two tests. In patients with TSH levels between 10 and 20 mIU/L, levothyroxine replacement therapy should be considered on a case-by-case basis, taking account of the patient’s wishes, expected benefits and TSH progression (64). Simultaneously, the levothyroxine therapeutic window is narrow, leading to overtreatment that is particularly common in older individuals (70). The guidelines recommended that higher serum TSH treatment targets may be appropriate, especially for the oldest old (patients >80 years) (28, 71).

The serum TSH RI should be consistent with the threshold for further clinical action. Therefore, a higher thyrotropin threshold for diagnosing and treating SHypo in the elderly seems reasonable. Using age-specific RIs, or even different intervals for different age groups of older adults, may result in older individuals with abnormal thyroid function being reclassified as normal (53, 54, 55, 59), and they may benefit from this reclassification (72).

## Conclusions

In this brief review, the current understanding of TSH changes with aging has been summarized. The TSH RIs should be established based on age, race, gender and iodine intake, with strict exclusion criteria in place. Due to the limited number and heterogeneity of older reference subjects in current studies, further research is needed. Overall, higher TSH levels and upper limits of RIs were observed in older individuals. Therefore, it seems reasonable to consider a higher TSH threshold for diagnosing and treating SHypo in the elderly.

## Declaration of interest

The authors declare that there is no conflict of interest that could be perceived as prejudicing the impartiality of the work reported.

## Funding

This work was supported by the Research Fund for Public Welfare, the National Health and Family Planning Commission of China (Grant No. 201402005), the National Natural Science Foundation of China (Grant No. 81970682) and the National Key Technologies R&D Program provided by the Ministry of Science and Technology of the People’s Republic of China (Project Grant No. 2022YFC3602300, Sub-project Grant No. 2022YFC3602303).
